# Effects of a newly developed potent orexin-2 receptor-selective antagonist, compound 1 m, on sleep/wakefulness states in mice

**DOI:** 10.3389/fnins.2014.00008

**Published:** 2014-01-31

**Authors:** Keishi Etori, Yuki C. Saito, Natsuko Tsujino, Takeshi Sakurai

**Affiliations:** Department of Molecular Neuroscience and Integrative Physiology, Faculty of Medicine, Institute of Medical, Pharmaceutical and Health Sciences, Kanazawa UniversityKanazawa, Japan

**Keywords:** orexin receptor antagonists, sleep, wakefulness, REM sleep, orexin

## Abstract

Orexins (also known as hypocretins) play critical roles in the regulation of sleep/wakefulness states by activating two G-protein coupled receptors (GPCRs), orexin 1 (OX_1_R) and orexin 2 receptors (OX_2_R). In order to understand the differential contribution of both receptors in regulating sleep/wakefulness states we compared the pharmacological effects of a newly developed OX_2_R antagonist (2-SORA), Compound 1 m (C1 m), with those of a dual orexin receptor antagonist (DORA), suvorexant, in C57BL/6J mice. After oral administration in the dark period, both C1m and suvorexant decreased wakefulness time with similar efficacy in a dose-dependent manner. While C1m primarily increased total non-rapid eye movement (NREM) sleep time without affecting episode durations and with minimal effects on REM sleep, suvorexant increased both total NREM and REM sleep time and episode durations with predominant effects on REM sleep. Fos-immunostaining showed that both compounds affected the activities of arousal-related neurons with different patterns. The number of Fos-IR noradrenergic neurons in the locus coeruleus was lower in the suvorexant group as compared with the control and C1m-treated groups. In contrast, the numbers of Fos-IR neurons in histaminergic neurons in the tuberomamillary nucleus and serotonergic neurons in the dorsal raphe were reduced to a similar extent in the suvorexant and C1m groups as compared with the vehicle-treated group. Together, these results suggest that an orexin-mediated suppression of REM sleep via potential activation of OX_1_Rs in the locus coeruleus may possibly contribute to the differential effects on sleep/wakefulness exerted by a DORA as compared to a 2-SORA.

## Introduction

A series of studies have suggested that loss of hypothalamic neurons producing orexin (orexin neurons) causes narcolepsy in humans and other mammalian species, showing that orexin plays an extremely important role in the regulation of sleep/wakefulness states, especially in the maintenance of wakefulness (Sakurai and Mieda, 2011). Because orexin is an arousal-promoting factor, it is reasonable to hypothesize that orexin receptor antagonists will be effective as drugs for the treatment of insomnia. Indeed, several orexin receptor antagonists with different pharmacological characteristics are under development as next generation sleep-inducing drugs.

A dual orexin receptor antagonist (DORA), almorexant (ACT-078573), blocks both OX_1_R and OX_2_R with similar potency (IC_50_ 16 and 15 nM, respectively). Almorexant was reported to shorten the time spent awake and maintain sleep in rats, dogs, and humans (Brisbare-Roch et al., 2007; Hoever et al., 2010). Almorexant significantly improved the primary parameter of sleep efficiency in humans (time spent sleeping while confined to bed during an 8 h period at night) in a dose-dependent manner. Almorexant decreased the latency to sleep onset and the number of wakefulness bouts after sleep onset. Importantly, almorexant not only changed these physiological sleep parameters, but also significantly improved subjective sleep quality. Effective or even higher doses of almorexant did not cause any significant negative effects on next-day performance (assessed by fine motor testing and mean reaction time). In addition, it was reported that rats administered a high dose of almorexant (300 mg/kg, p.o.) were fully capable of spatial and avoidance learning (Dietrich and Jenck, 2010). Notably, almorexant was well tolerated with no sign of cataplexy, suggesting that acute, short-lived, intermittent temporary blockade of orexin receptors will not result in a narcolepsy-like phenotype (Neubauer, 2010).

Phase III clinical trials of suvorexant (MK-4305), a DORA developed by Merck & Co., for the modulation of sleep have been completed (Cox et al., 2010). Suvorexant is a potent DORA with excellent potency in cell-based calcium mobilization assays (OX_1_R IC_50_ = 50 nM, OX_2_R IC_50_ = 56 nM) (Winrow and Renger, 2013). Recent studies showed that patients taking the drug fell asleep faster and slept longer than those on placebo, with no major adverse effects (Hopkins, 2012; Mieda and Sakurai, 2013). Suvorexant is expected to be available for clinical use in 2014.

Recently, administration of a selective OX_2_R antagonist (2-SORA), JNJ-10397049, in rats was also shown to decrease the latency to persistent sleep and to increase NREM sleep time more potently than did almorexant (Dugovic et al., 2009), while a selective OX_1_R antagonist, SB-408124, had no effect on sleep parameters. Rather, SB-408124 attenuated the sleep-promoting effects of the OX_2_R antagonist when simultaneously administered, possibly by increasing dopamine release in the prefrontal cortex. However, the effectiveness of DORA and 2-SORA is a controversial issue, because another report suggested that almorexant is more effective for sleep promotion than is antagonism of either receptor alone (Morairty et al., 2012). Further research using selective antagonists with different pharmacological characteristics is required to reach a conclusion on the effectiveness, advantages and disadvantages of these compounds.

In this study, we compared the effects of a newly-developed potent OX_2_R-selective antagonist (2-SORA), Compound 1m (C1m), with those of suvorexant. C1m showed potent OX_2_R antagonistic activity (IC_50_ 27 nM) and good selectivity against OX_1_R (IC_50_ 3000 nM, determined by cell-based calcium mobilization assay using receptor-expressing cells). C1m is an amphiphilic molecule simultaneously possessing high water solubility and lipophilicity, to which its good oral availability is attributable (Fujimoto et al., 2011). Furthermore, this compound showed excellent metabolic stability in human and rat liver microsomes (Fujimoto et al., 2011).

We found C1m had comparable efficacy to that of suvorexant in increasing NREM sleep time, but showed little effect on REM sleep amount, while suvorexant significantly increased REM sleep. Suvorexant induced longer NREM and REM sleep episode durations as compared with C1m. Suvorexant and C1m affected the number of Fos-positive monoaminergic neurons in the brain stem with differential patterns. These results suggest differential roles of OX_1_R and OX_2_R in sleep/wakefulness regulation.

## Materials and methods

### Animals

All mice used in this study had a C57B6/J genetic background and were 12–15 weeks of age and weighed 25–30 g. They were fed *ad libitum* and housed under conditions where temperature (22°C) and humidity were controlled with a 12-h light/dark cycle (lights on at 8:45 a.m., off at 8:45 p.m.). All experimental procedures were approved by the Animal Experimental and Use Committee of Kanazawa University (AP-132649) and were in accordance with NIH guidelines. All efforts were made to minimize animal suffering and discomfort and to reduce the number of animals used.

### Substances and administration

C1m, a novel potent 2-SORA, was provided by Takeda Pharmaceutical, Co., Ltd. (Japan) (Fujimoto et al., 2011). A DORA, [(7R)-4-(5-chloro-1,3-benzoxazol-2-yl)-7-methyl-1,4-diazepan-1-yl][5-methyl-2-(2H-1,2,3-triazol-2-yl)phenyl]methanone (suvorexant) was synthesized according to a previously reported procedure (Cox et al., 2010) (lot # 130301, NARD Institute, Amagasaki, Japan). Drugs were suspended in 1% methylcellulose (Sigma) and administered to mice *per os* using a disposable feeding needle (Fuchigami Kikai, Japan) at Zeitgeber time (ZT) 12 or ZT0.

### Sleep recordings

Mice were anesthetized with sodium pentobarbital, and an electrode was implanted for EEG/EMG recording. Four holes were drilled in the skull, and the arms of the electrode for EEG were implanted at sites approximately 2 mm anterior, ± 2 mm lateral, and 2 mm posterior to the bregma. EMG recording wires made of stainless steel were inserted into the neck muscles bilaterally. Each electrode was fixed rigidly to the skull with dental cement (ESPE Ketac-Cem). After the recovery period (5–6 days after surgery), mice were moved to a recording cage placed in an electrically shielded and sound-attenuated room. The implanted electrode of each mouse was connected to a cable for signal output. They were allowed to move freely with access to food and water *ad libitum*. Signals were amplified through an amplifier (AB-611J, Nihon Koden, Tokyo) and digitally recorded on a computer using EEG/EMG recording software (Vital recorder, Kissei Comtec). Mice were put in recording cages for at least 7 days to allow them to adapt to the recording conditions prior to any EEG/EMG recording session. Following the acclimatization period, 1% methylcellulose as control, C1m and suvorexant were orally administered to mice on separate experimental days with an interval of at least 3 days. Each dose of drugs was explored in different groups of mice (*n* = 5–9/group). We did not use mice repeatedly, in order to avoid the influences of repeated administration procedures and residual effects of drugs. EEG/EMG data for 24 h following drug administration were evaluated as previously described (Hara et al., 2001).

### Statistical analysis

Data were expressed as mean ± s.e.m. Two-Way analysis of variance (ANOVA) followed by Bonfferoni correction as a *post-hoc* test or Student's *t*-test using GraphPad Prism 6.01 was used for comparison among the various treatment groups. Differences were considered significant at *p* < 0.05.

### Immunohistochemistry

Mice were anesthetized deeply and perfused with 60 ml ice-cold phosphate buffer saline (PBS) and 40 ml ice-cold 4% paraformaldehyde (PFA) in 0.1 M phosphate buffer 2 h after drug administration at ZT12. The brain was removed and immersed in 4% PFA for 24 h at 4°C, and then in 30% sucrose in 0.1 M PBS for 2 days. The brain was then frozen quickly in embedding solution (Sakura Finetek Co., Ltd., Tokyo, Japan) and cut into coronal sections (30-μm thick) using a cryostat (HM 505E, Micron, Walldorf, Germany). Coronal brain sections were washed three times for 10 min in 0.1 M PBS containing 1% bovine serum albumin and 0.25% Triton-X-100 (PBS-BX). To detect Fos-like immunoreactivity (IR) in orexin-expressing neurons, the sections were incubated overnight at 4°C with guinea pig anti-orexin antibody (1:500) and rabbit anti-cFos antibody Ab-5 (Calbiochem, 1:10,000). After washing three times with PBS-BX, tissue was incubated for 1 h with Alexa Fluor 594-goat anti-guinea pig IgG (Molecular Probes, 1:800) and Alexa Fluor 488-goat anti-rabbit IgG (Molecular Probes, 1:800) and then washed three times again. The sections were mounted on glass slides and cover-slipped, and the slides were examined by laser-confocal microscopy (Olympus FV10i).

To detect Fos-IR in serotonergic, histaminergic, and noradrenergic neurons, coronal brain sections were incubated with mouse anti-tryptophan hydroxylase (TPH) antibody (Santa Cruz Biotech, 1:200), guinea pig anti-histidine decarboxylase (HDC) antibody (PROGEN Biotechnik Gmbh, 1:4000), or mouse anti-tyrosine hydroxylase (TH) antibody (Santa Cruz Biotech, 1:2000), respectively, with rabbit anti-Fos antibody Ab-5 (Calbiochem, 1:10,000). As a second antibody, Alexa Fluor 594-goat anti-mouse IgG (Molecular Probes, 1:800), Alexa Fluor 594-goat anti-guinea pig IgG (Molecular Probes, 1:800), or Alexa Fluor 488-goat anti-rabbit IgG (Molecular Probes, 1:800) was used.

## Results

### C1m increased NREM sleep time without affecting REM sleep time

**Figure 1:**
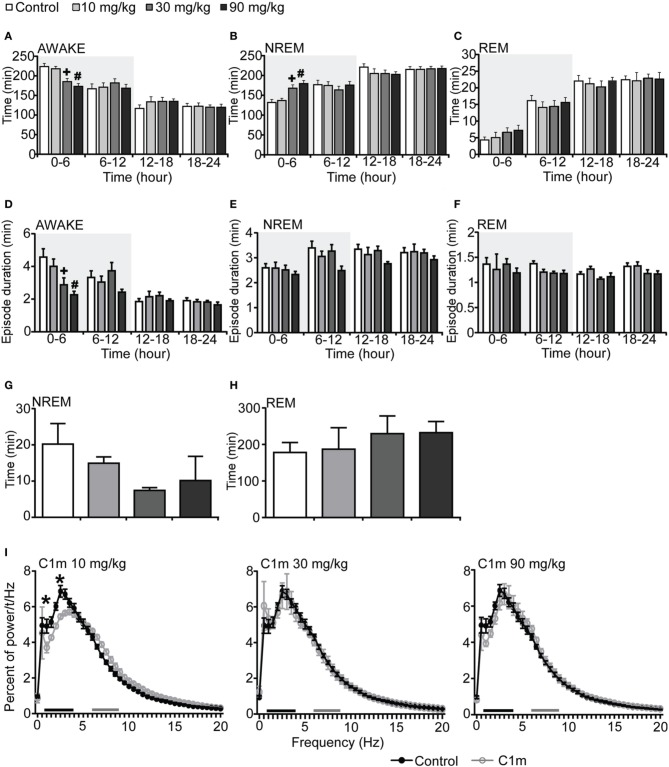
**Effects of C1m on basal sleep/wakefulness states in C57BL/6 mice (*n* = 6–9/group) after administration at start of dark period**. C1m (10, 30, 90 mg/kg) and methylcellulose as control were administered *per os* at the start of the light period (*t* = 0, *ZT* = 12). **(A–C)** Total time spent in wakefulness **(A)**, NREM sleep **(B)**, and REM sleep **(C)** in 6 h time windows over 24 h. **(D–F)** Mean duration of wakefulness **(D)**, NREM sleep **(E)** and REM sleep **(F)** in 6 h time windows over 24 h. Data for the dark and light periods are displayed with light gray and white backgrounds, respectively. **(G,H)** Latency to NREM sleep (time to appearance of first NREM sleep after administration) **(G)** and REM sleep latency (time to appearance of first REM sleep after administration) **(H)** during dark period. Results are expressed in minutes and presented as mean ± s.e.m. **(I)** EEG power density during NREM sleep for 3 h after administration shown as mean percentage of total EEG power. The delta range (0.75–4 Hz) is indicated by the black bar and the theta range (6–9 Hz) by the gray bar. ^*^p < 0.05 for 10 mg/kg C1m, ^+^*p* < 0.05 for 30 mg/kg C1m, ^#^*p* < 0.05 for 90 mg/kg C1m vs. control.

To examine the effect of C1m on sleep/wakefulness states, we administered it orally to mice at the start of the dark period. Mice administered C1m (30 and 90 mg/kg) showed significantly shorter wakefulness time as compared with vehicle-administered mice for 6 h after administration (Figure 1A, Figure S1A). Wakefulness time for 6 h post-administration was 17.2 and 22.6% shorter in the 30 and 90 mg/kg C1m groups, respectively, as compared to that in the control group [*F*_(3, 26)_ = 9.55, *p* < 0.001] (Figure 1A). Hourly analysis suggested that the effect lasted for 5 h (Figure S1A). The decrease of wakefulnes was accompanied by a dose-dependent increase of NREM sleep time (Figure 1B, Figure S1B), which was significant during the 6 h after administration [*F*_(3, 26)_ = 8.54, *p* < 0.01 for 30 mg/kg, *p* < 0.001 for 90 mg/kg] (Figure 1B). Importantly, no significant difference in total REM sleep time was observed between the C1m and vehicle groups, although there was a weak tendency for C1m to increase total REM sleep tine (Figure 1C, Figure S1C). The C1m-administered group showed shorter wakefulness episode durations as compared with the vehicle-treated group [*F*_(3, 26)_ = 5.39, *p* < 0.05 for 30 mg/kg, *p* < 0.01 for 90 mg/kg] (Figure 1D). NREM and REM sleep episode durations were not affected by C1m (Figures 1E,F, Figures S1E,F).

Latency to NREM sleep onset after administration of C1m show a tendency to be shorter than that in the control group, but the difference was not statistically significant (Figure 1G). Latency to the onset of REM sleep after administration was not significantly different between the C1m- and vehicle-administered groups (Figure 1H). The power density of EEG in the C1m-administered group (30 and 90 mg/kg) showed no difference from that in the vehicle-administered group, specifically in regard to NREM delta power (0.5–4 Hz) (Figure 1I). However, we observed decrease in slow wave power in the low dose (10 mg/kg) group [*F*_(40, 246)_ = 2.547, *p* < 0.01 for 1 Hz, *p* < 0.001 for 2.5 Hz] (Figure 1I).

**Figure 2:**
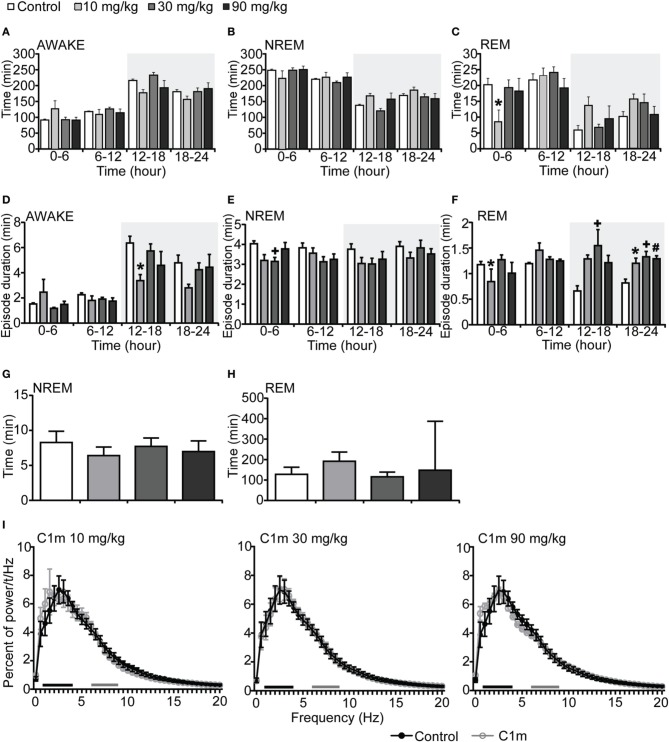
**Effects of C1m on basal sleep/wakefulness states in C57BL/6 mice (*n* = 6–7/group) after administration at start of light period**. C1m (10, 30, 90 mg/kg) and methylcellulose as control were administered *per os* at the start of the light period (*t* = 0, *ZT* = 0). **(A–C)** Total time spent in wakefulness **(A)**, NREM sleep **(B)**, and REM sleep **(C)** in 6 h time windows over 24 h. **(D–F)** Mean duration of wakefulness **(D)**, NREM sleep **(E)** and REM sleep **(F)** in 6 h time windows over 24 h. Data for the dark and light periods are displayed with light gray and white backgrounds, respectively. **(G,H)** Latency to NREM sleep (time to appearance of first NREM sleep after administration) **(G)** and REM sleep latency (time to appearance of first REM sleep after administration) **(H)** during light period. Results are expressed in minutes and presented as mean ± s.e.m. (**I)** EEG power density during NREM sleep for 3 h after administration shown as mean percentage of total EEG power. The delta range (0.75–4 Hz) is indicated by the black bars and the theta range (6–9 Hz) by the gray bars.^*^p < 0.05 for 10 mg/kg C1m, ^+^*p* < 0.05 for 30 mg/kg C1m, ^#^*p* < 0.05 for 90 mg/kg C1m vs. control.

We next administered C1m just prior to the start of the light period (ZT0). The total wakefulness and NREM sleep times were not significantly different between the C1m and vehicle groups during the light period (Figures 2A,B, Figures S2A,B). REM sleep time for 6 h after the administration in the low dose C1m group (10 mg/kg) was shorter as compared with the control group, suggesting that low dose of C1m rather shortens REM sleep time (Figure 2C, Figure S2C). Wakefulness episode duration was also not affected by C1m (Figure 2D). However, NREM and REM sleep episode durations were significantly shorter in the C1m groups (30 and 10 mg/kg) than in the control group (Figures 2E,F, Figures S2E,F). Latencies to NREM and REM sleep onset after administration of C1m were not significantly different from that in the control group (Figures 2G,H). The power density of EEG in the C1m-administered groups showed no difference from that in the vehicle-administered group in NREM sleep (Figure 2I).

### Suvorexant decreased wakefulness time and increased both NREM and REM sleep times

**Figure 3:**
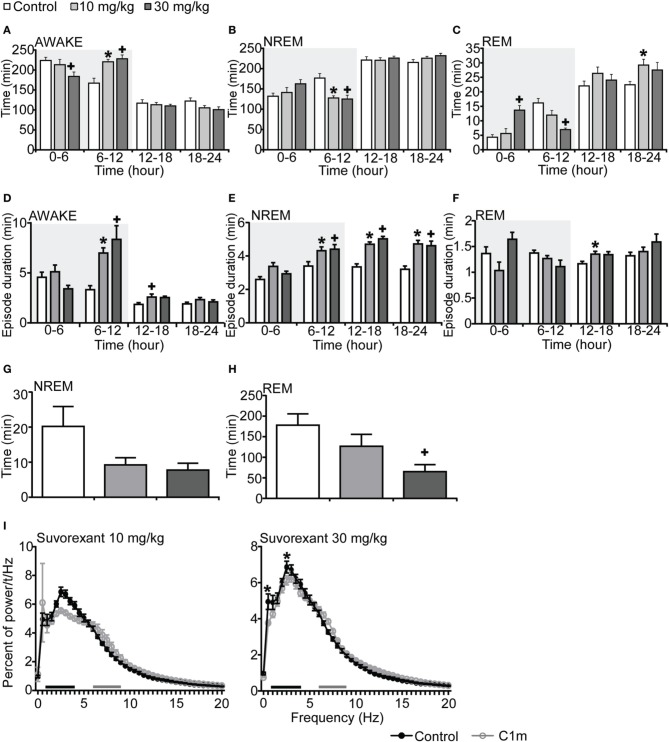
**Effects of DORA, suvorexant, on basal sleep/wakefulness states in C57BL/6 mice (*n* = 7–9/group) after administration at ZT12**. Suvorexant (10 and 30 mg/kg) and methylcellulose as control were administered *per os* at the start of the dark period (*t* = 0, ZT12). **(A–C)** Total time spent in wakefulness **(A)**, NREM sleep **(B)** and REM sleep **(C)** in 6 h time windows over 24 h. **(D–F**) Mean duration of wakefulness **(D)**, NREM sleep **(E)** and REM sleep **(F)** in 6 h time windows over 24 h. Data for the dark and light periods are displayed with light gray and white backgrounds, respectively. **(G,H)**. Latency to NREM sleep (time to appearance of first NREM sleep after administration) **(G)** and REM sleep latency (time to appearance of first REM sleep after administration) **(H)** during dark period. Results are expressed in minutes and presented as mean ± s.e.m. **(I)** EEG power density during NREM sleep for 3 h after administration shown as mean percentage of total EEG power. The delta range (0.75–4 Hz) is indicated by the black bars and the theta range (6–9 Hz) by the gray bars. ^*^p < 0.05 for 10 mg/kg suvorexant, ^+^*p* < 0.05 for 30 mg/kg suvorexant vs. control.

To compare the effect of C1m with that of a DORA, we also examined the effect of suvorexant, a DORA, on sleep/wakefulness states of mice under the same recording condition. Mice administered suvorexant (30 mg/kg) at the start of the dark period showed a significantly shorter wakefulness time as compared with vehicle-administered mice for 6 h after administration (Figure 3A, Figure S3A). The wakefulness time for 6 h post-administration of suvorexant (30 mg/kg) was shortened by 17.8% [*F*_(2, 20)_ = 3.74, *p* < 0.05] (Figure 3A). This effect was accompanied by increases of both NREM and REM sleep time (Figure 3C, Figures S3B,C). Significant differences were also observed in the latter half of the dark period; wakefulness time was rather longer and NREM and REM sleep times were shorter in the suvorexant group than in the control group in this time window (Figures 3A–C, Figures S3A–C). These effects are likely to be the rebound of wakefulness due to homeostatic mechanisms controlling the amount of sleep.

Episode durations of wakefulness and NREM sleep in the suvorexant group for 6 h after administration were not different from those in the control group (Figures 3D,E, Figures S3D,E). However, there were significant differences in these parameters in the latter half of the dark period between the suvorexant and control groups [Wakefulness episodes: *F*_(2, 20)_ = 10.58, *p* < 0.01 for 10 mg/kg, *p* < 0.001 for 30 mg/kg, Figure 3D] [NREM sleep episodes: *F*_(2, 20)_ = 4.86, *p* < 0.05 for 10 mg/kg, *p* < 0.05 for 90 mg/kg, Figure 3E]. The longer episode duration of NREM sleep in the suvorexant group continued in the subsequent light period. These observations suggest that suvorexant consolidates both wakefulness and NREM sleep episodes. REM sleep episode duration was not significantly affected by suvorexant for 12 h after administration (Figure 3F). However, hourly analysis showed that high dose (30 mg/kg) suvorexant increased REM sleep duration for several hours (Figure S3F). Latency to NREM sleep onset after administration of C1m showed a tendency to be shorter than that in the control group, although the difference was not statistically significant (Figure 3G). REM sleep latency after administration was shorter in the 30 mg/kg group than in the vehicle group during the dark period [*F*_(2, 20)_ = 4.92, *p* < 0.05] (Figure 3H).

The power density of EEG in the suvorexant-administered group (30 mg/kg) showed slightly, but significantly larger percent of 0.5 and 2.5 Hz component [*F*_(40, 246)_ = 2.539, *p* < 0.0001 for 0.5 Hz, *p* < 0.05 for 2.5 Hz] (Figure 3I).

**Figure 4:**
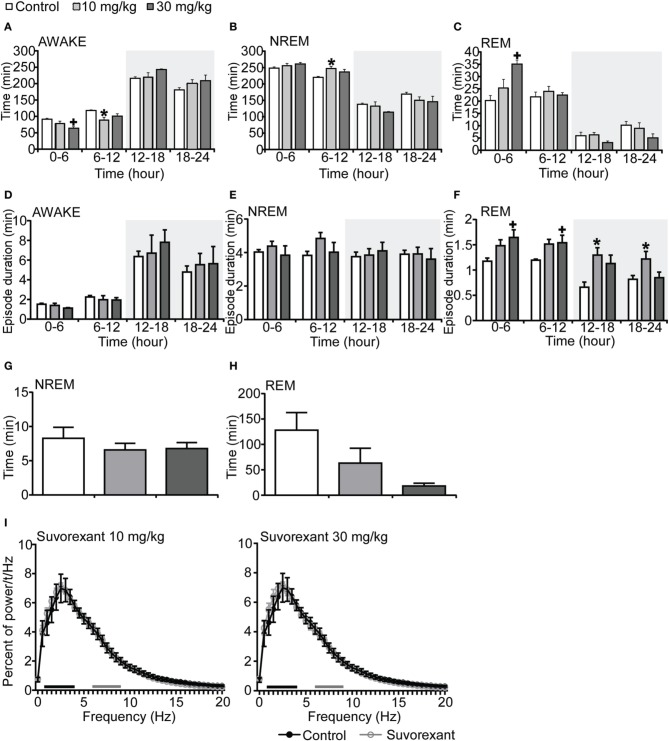
**Effects of suvorexant on basal sleep/wakefulness states in C57BL/6 mice (*n* = 5–7/group) after administration at start of light period**. Suvorexant (10 and 30 mg/kg) and methylcellulose as control were administered *per os* at the start of the recording (*t* = 0, ZT0). **(A–C)** Total time spent in waking **(A)**, NREM sleep **(B)** and REM sleep **(C)** in 6 h time windows over 24 h. **(D–F)** Mean duration of wakefulness **(D)**, NREM sleep **(E)**, and REM **(F)** sleep in 6 h time windows over 24 h. Data for the dark and light periods are displayed with light gray and white backgrounds, respectively. **(G,H)** Latency to NREM sleep (time to appearance of first NREM sleep after administration) **(G)** and REM sleep latency (time to appearance of first REM sleep after administration) **(H)** during light period. Results are expressed in minutes and presented as mean ± s.e.m. **(I)** EEG power density during NREM sleep for 3 h after administration shown as mean percentage of total EEG power. The delta range (0.75–4 Hz) is indicated by the black bars and the theta range (6–9 Hz) by the gray bars. ^*^p < 0.05 for 10 mg/kg suvorexant, ^+^*p* < 0.05 for 30 mg/kg suvorexant vs. control.

Mice administered suvorexant (30 mg/kg) at the start of light period showed a shorter wakefulness time and longer REM sleep time after administration as compared with vehicle-administered mice (Figures 4A–C, Figures S4A–C). During the light phase, suvorexant showed an effect on wakefulness for 1 h and on REM sleep for 3 h (Figures S4A–C). Wakefulness and NREM sleep episode durations were not affected by suvorexant administration (Figures 4D,E). However, REM sleep episode duration was longer in the suvorexant group. This effect lasted for 24 h (Figure 4F). NREM and REM sleep latencies were not different statistically between the suvorexant and vehicle groups (Figure 4H). The power density of EEG in the suvorexant-administered group showed no difference from that in the vehicle-administered group (Figure 4I).

### Comparison of effects of C1m vs. suvorexant

**Figure 5:**
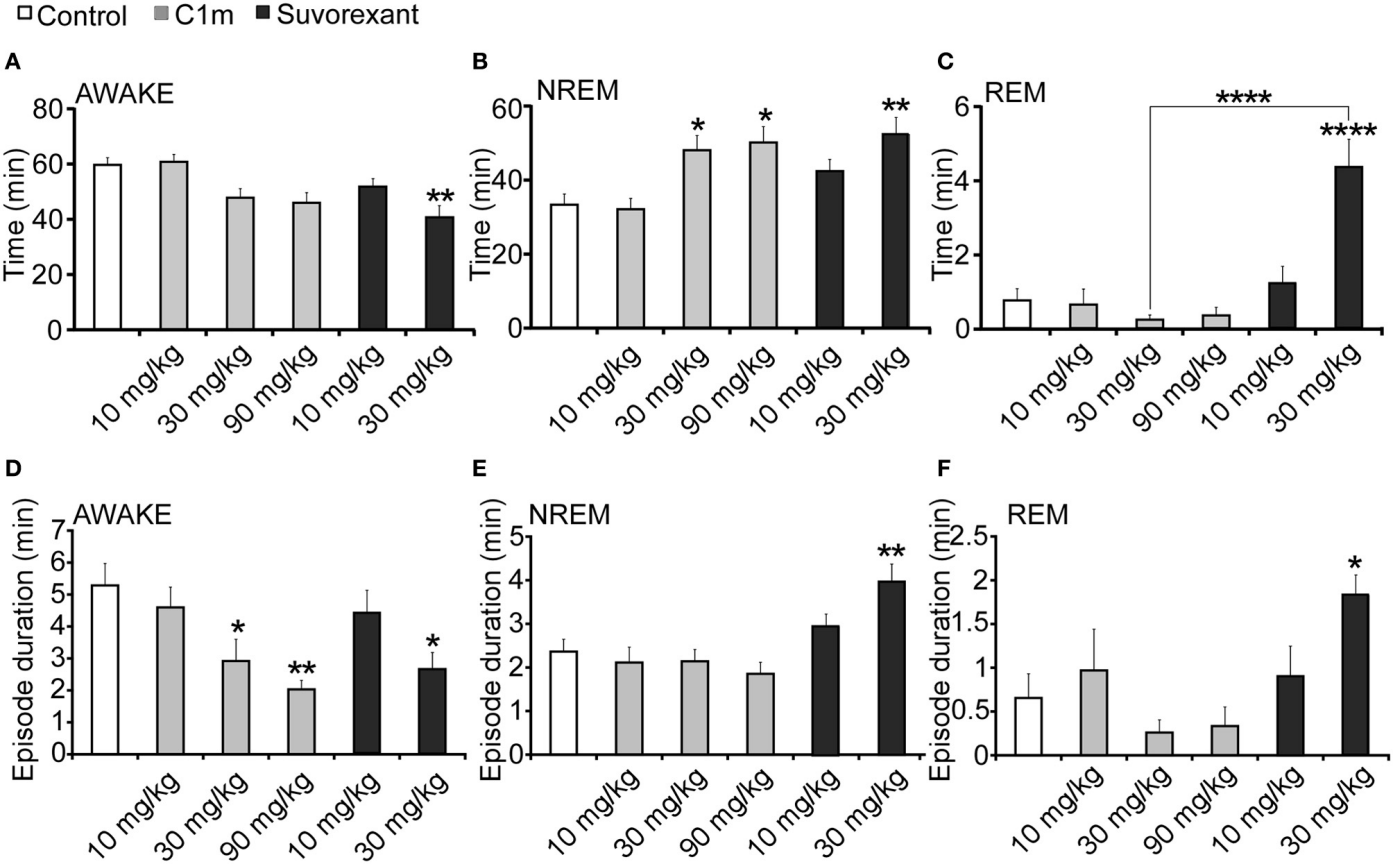
**Total time (A–C) and average episode duration (D–F) of awake (A,D), NREM sleep (B,E) and REM sleep (C,F) states for the first 2 h after administration of suvorexant or C1m at ZT12, compared with vehicle-administered group**. Data are expressed as percentage and presented as mean ± s.e.m. (*n* = 6–9/group). ^*^p < 0.05; ^**^p < 0.01 and ^****^p < 0.0001.

Considering the differences in the effective time periods of action of both compounds, we also compared the effects of these drugs in the time window of 2 or 3 h after administration (Figure 5, Table 1).

**Table 1:** **Total number of state transitions after administration of Compound 1 m (10, 30, and 90 mg/kg) and Suvorexant (10 and 30 mg/kg) in wild-type mice at ZT 12**.

	**Vehicle**	**Compound 1 m**			**Suvorexant**	
	**(***n*** = **6**)**	**10 mg/kg (***n*** = **6**)**	**30 mg/kg (***n*** = **9**)**	**90 mg/kg (***n*** = **6**)**	**10 mg/kg (***n*** = **7**)**	**30 mg/kg (***n*** = **7**)**
W → NR	25.2 (2.8)	28.0 (5.0)	41.8 (6.8)^*^	44.7 (5.3)^*^	22.7 (2.4)	30.4 (1.7)
W → R	0	0	0	0	0	0
NR → W	23.9 (2.9)	26.8 (5.3)	40.7 (7.0)	43.6 (5.5)^*^	20.4 (2.8)	25.3 (2.1)
NR → R	1.2 (0.4)	0.8 (0.3)	1.0 (0.3)	0.9 (0.5)	2.1 (0.9)	4.4 (0.8)^**^
R → W	1.2 (0.4)	0.8 (0.3)	1.0 (0.3)	0.9 (0.5)	2.1 (0.9)	4.3 (0.8)^**^
R → NR	0	0	0	0	0	0

*Values (means ± s.e.m.) are calculated for 3 h after administratrion at ZT 12. W, NR, and R represents wake, NREM sleep, and REM sleep, respectively*.

* P<0.05 and

** *P<0.01 for each dose vs. control, one-way ANVA followed by Bonfferoni correction as post-hoc test*.

For 2 h after administration at ZT12, both C1m and suvorexant increased NREM sleep time [*F*_(5, 38)_ = 5.33, *p* < 0.05 for C1m 30 mg/kg, *p* < 0.05 for C1m 90 mg/kg, *p* < 0.01 for suvorexant 30 mg/kg] (Figure 5B). There was a major difference in the effect on REM sleep time: C1m showed little effect on REM sleep time even at a high dose (90 mg/kg), while suvorexant (30 mg/kg) markedly increased REM sleep time [*F*_(5, 38)_ = 14.06, *p* < 0.0001] (Figure 5C). There was also a difference in the effects of both compounds on each episode duration. Both C1m and suvorexant shortened the wakefulness episode duration in a dose-dependent manner [*F*_(5, 38)_ = 4.08, *p* < 0.05 for C1m 30 mg/kg, *p* < 0.01 for C1m 90 mg/kg, *p* < 0.05 for suvorexant 30 mg/kg] (Figure 5D). C1m did not change the NREM sleep or REM sleep episode durations (Figures 1E,F), whereas suvorexant (30 mg/kg) increased both the REM [*F*_(5.38)_ = 3.76, *p* < 0.05] and NREM sleep [*F*_(5, 38) = 5.74_, *p* < 0.01] episode durations (Figures 5E,F).

While C1m increased the transition numbers of both wakefulness to NREM sleep and NREM sleep to wakefulness, suvorexant did not show any effects on these parameters (Table 1). On the other hand, suvorexant increased NREM to REM sleep and REM to wakefulness transitions, while C1m did not influence them (Table 1).

### Effects of C1m and suvorexant on activity of orexinergic, noradrenergic, serotonergic, and histaminergic neurons

To define the effects of C1m and suvorexant on activity of arousal-related neurons, we examined Fos-like immunoreactivity (Fos-IR) in orexin neurons, noradrenergic neurons in the locus coeruleus (LC), serotonergic neurons in the dorsal raphe (DR) nucleus, and histaminergic neurons in the tuberomammillary nucleus (TMN). Since Fos-IR generally reflects the activity of neurons for 60–90 min before the time of fixation, we killed mice 2 h after drug administration. Mice were administered drugs at ZT12, and killed at ZT14.

**Figure 6:**
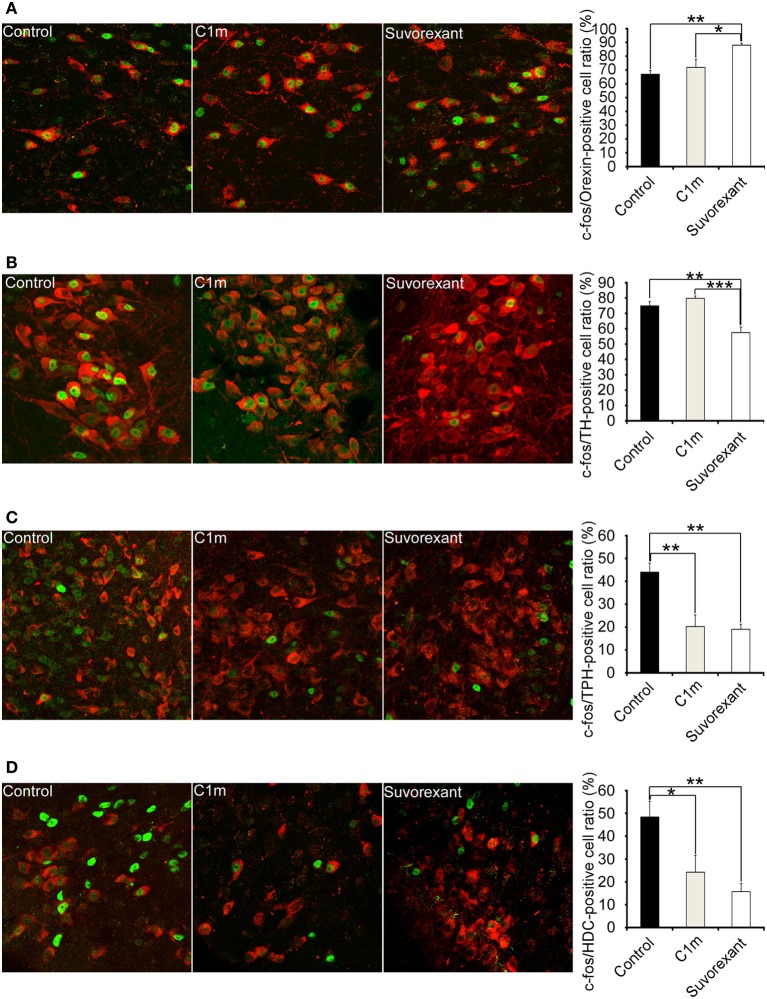
**Number of Fos-positive neurons at 2 h after administration of vehicle and drugs (30 mg/kg C1m and suvorexant) at ZT12 in C57BL/6 mice (*n* = 6/group)**. Typical images of Fos expression in orexin-IR cells in lateral hypothalamic area **(A)**, TH-IR cells in LC **(B)**, TPH-IR cells in DR **(C)**, and HDC-IR cells in TMN **(D)**. Fos-IR nucleus-positive cells are calculated as the percentage of orexin-IR cells (red) with a Fos-IR nucleus (green) in all orexin neurons, TH-IR cells (red) with a Fos-IR nucleus (green) in all TH-IR cells, TPH-IR cells (red) with a Fos-IR nucleus (green) in all TPH-IR cells, and HDC-IR cells (red) with a Fos-IR nucleus (green) in all HDC-IR cells. Data are expressed as percentage and presented as mean ± s.e.m. ^*^p < 0.05, ^**^p < 0.01 and ^***^p < 0.001. TH, tryptophane hydroxylase; LC, locus coeruleus; TPH, tryptophan hydroxylase; HDC, histidine decarboxylase; TMN, tuberomammillary nucleus.

Whereas the numbers of Fos-positive orexin neurons and noradrenergic neurons were not affected by C1m, suvorexant significantly increased the number of Fos-positive orexin neurons (Control, 67.0 ± 2.5%; suvorexant, 88.0 ± 1.3%, [*F*_(2, 15) = 8.90, *p* < 0.01]_ and decreased the number of Fos-positive noradrenergic neurons [Control, 74.9 ± 2.8%; suvorexant, 57.5 ± 3.6%, *F*_(2, 15)_ = 17.65, *p* < 0.01)] (Figures 6A,B). C1m (30 mg/kg) administration significantly decreased the numbers of Fos-positive serotonergic neurons [Control, 44.1 ± 3.9%; C1m, 20.3 ± 5.0%, *F*_(2, 15)_ = 13.21, *p* < 0.01] and histaminergic neurons [Control, 48.7 ± 6.9%; C1m, 24.3 ± 7.4%, *F*_(2, 15)_ = 7.57, *p* < 0.05] (Figures 6C,D). Similarly, suvorexant (30 mg/kg) significantly decreased the numbers of Fos-positive serotonergic neurons [Control, 44.1 ± 3.9%; suvorexant, 19.1 ± 2.2%, *F*_(2, 15)_ = 13.21, *p* < 0.01] and histaminergic neurons [Control, 48.7 ± 6.9%; suvorexant, 15.8 ± 3.5%, *F*_(2, 15)_ = 7.57, *p* < 0.01] (Figures 6C,D).

## Discussion

Orexin receptor antagonists, especially DORAs, are under development as next generation drugs for treating insomnia. Precise knowledge about differential roles of the two orexin receptors would be beneficial for application of orexin agonists/antagonists as treatment for various diseases. It has been thought that OX_2_R plays a pivotal role in the maintenance of wakefulness, based on the phenotype of receptor-deficient mice and pharmacological studies using these mice (Sakurai and Mieda, 2011). *OX_2_R* knockout mice show characteristics of narcolepsy (Willie et al., 2003), while *OX_1_R* knockout mice show an almost normal sleep-wake cycle (Willie et al., 2001). However, the phenotype of *OX_2_R* knockout mice is less severe than that found in *prepro-orexin* knockout mice and double receptor knockout mice. Especially, *OX_2_R* knockout mice are almost 33 times less affected by cataplexy and direct transitions to REM sleep from an awake state as compared with orexin ligand knockout mice (Willie et al., 2003). Furthermore, the effects of orexin A on wakefulness and NREM sleep were significantly attenuated in *OX_2_R*^−/−^ mice as compared with wild-type mice and *OX_1_R*^−/−^ mice, although *OX_1_R*^−/−^ mice showed a slightly impaired response (Mieda et al., 2011). Notably, suppression of REM sleep by orexin-A administration was similarly attenuated in both *OX_1_R*^−/−^ and *OX_2_R*^−/−^ mice, suggesting a comparable contribution of the two receptors to REM suppression. These observations suggest that although the OX_2_R-mediated pathway has a pivotal role in the promotion of wakefulness, OX_1_R also has additional effects on sleep/wakefulness regulation, especially in the inhibitory regulation of REM sleep.

In this study, we examined the effect of a novel 2-SORA, C1m, in mice. We found that C1m (30 and 90 mg/kg) significantly reduced wakefulness time along with an increase in NREM sleep time for 5 h after administration at the start of the dark period (Figures 1A,B). The efficacy of C1m in increasing NREM sleep time was comparable (Figure 5B) or even stronger (Figures 1B, 3B) than that of suvorexant, depending on the observation time. This indicates that the sole blockade of OX_2_R is sufficient to increase NREM sleep time. This is consistent with a previous pharmacological study suggesting that wakefulness/NREM sleep transition depends primarily on OX_2_R (Mieda et al., 2011). However, while suvorexant increased the NREM sleep episode duration, C1m did not (Figure 5E). Likewise, C1m increased the number of transitions between wakefulness and NREM sleep, while suvorexant did not (Table 1). These findings suggest that OX_1_R might have additional effects of increasing wakefulness, and blocking of OX_1_R along with OX_2_R blockade further consolidates NREM sleep. This is again consistent with our previous study showing that ICV orexin-A still increased wakefulness in *OX_2_R*^−/−^ mice (Mieda et al., 2011).

Both C1m and suvorexant showed greater sleep-promoting effects in mice during the dark period, in which orexin neurons fire rapidly (Lee et al., 2005), than during the light period. Still, whereas the administration of C1m just prior to onset of the light phase had only minimal effects on wakefulness, suvorexant (30 mg/kg) was able to significantly decreased wakefulness time (Figure 4A) and increase REM sleep time also in the light period (Figure 4C, Figure S4C). These observations suggest that DORAs might have a more powerful impact on sleep/wakefulness states especially during the light period as compared with 2-SORA. This could suggest a role of OX_1_R in gating of the transition from NREM sleep to wakefulness during the resting period. The impact of 1 h of suvorexant on wakefulness time when administered during the day (Figure S3A) could be due to more rapid onset of action of suvorexant, because administration *per se* has a stimulant effect, and only a compound with a rapid onset of action could show efficacy at that moment.

We observed an increase of wakefulness in the 6–12 h time window after the administration of suvorexant at ZT12 (Figure 3A). However, we did not find a rebound increase of wakefulness in the C1m-administered group (Figure 1A). This difference is likely the result of the different time periods where both compounds exert biological activity. Alternatively, blocking OX_1_R might lead to more profound wakefulness rebound after the time of sleep-induction. This mechanism should be addressed in future studies.

*Orexin^−/−^* and *OX_2_R*^−/−^ mice show sleep fragmentation during the dark period, which is accompanied by a shorter NREM episode duration during the dark period (Chemelli et al., 1999; Willie et al., 2003). Although C1m increased the frequency of transitions between wakefulness and NREM sleep states (Table 1), it did not significantly shorten NREM sleep episode duration. Suvorexant increased the NREM sleep duration and decreased the frequency of transitions between NREM and REM sleep when it was administered in the dark period (Figure 5E). The increase in duration of NREM and REM sleep by DORAs is consistent with the results of previous studies (Winrow et al., 2012). These observations suggest that acute pharmacological blockade of OX_2_R or both receptors increases sleep time, but does not induce sleep/wakefulness fragmentation, one of the important characteristics of narcolepsy. These observations suggest that the sleep/wakefulness fragmentation in narcolepsy might be due to chronic compensatory processes in narcoleptic animals resulting from chronic deficiency of orexin signaling (Tsujino et al., 2013).

OX_1_R and OX_2_R are distributed differently in the brain. Histaminergic neurons in the TMN, which strongly express OX_2_R, are thought to play an important role in the arousal-promoting effect of orexin, because the effect of ICV orexin-A administration is markedly attenuated by the histamine H_1_ receptor antagonist pyrilamine and is absent in H_1_ histamine receptor knockout mice (Huang et al., 2001; Yamanaka et al., 2002). Mochizuki et al. produced a mouse model in which a loxP-flanked gene cassette disrupts production of OX_2_R, but normal OX_2_R expression can be restored by Cre recombinase (Mochizuki et al., 2011). They showed that targeted Cre expression, i.e., focal restoration of OX_2_R expression, in the TMN and adjacent regions abrogated fragmentation of wakefulness in this mouse model, suggesting that the orexin signaling mediated by OX_2_R in the TMN and/or its surrounding area in the posterior hypothalamus is sufficient to prevent sleepiness caused by systemic OX_2_R deficiency. However, orexins probably promote arousal through many redundant systems because optogenetic activation of orexin neurons still promotes wakefulness in mice lacking histamine (Carter et al., 2009), and mice lacking both OX_1_R and histamine H_1_ receptors demonstrate no abnormality of sleep/wakefulness (Hondo et al., 2010). Our present study further suggests an additional role of OX_1_R in promoting and maintaining wakefulness, and a relatively large impact on REM sleep amount.

To gain an insight into the mechanisms by which both compounds affect sleep/wakefulness states, we examined the effects of the compounds on the number of Fos-IR neurons in orexin-target areas (Figure 6). Compounds were administered at the start of the dark period. We found that the number of Fos-IR noradrenergic neurons in the LC was lower in the suvorexant group as compared with the control and C1m-treated groups (Figure 6B). This is consistent with the fact that noradrenergic neurons in the LC exclusively express OX_1_R (Mieda et al., 2011). The number of Fos-IR serotonergic neurons in the DR was similarly lower in the suvorexant and C1m groups than in the control group (Figure 6C). This suggests that orexin mainly excites serotonergic neurons through activation of OX_2_R, although these cells also express OX_1_R (Mieda et al., 2011). We observed that the number of Fos-IR neurons in histaminergic neurons in the TMN was lower in both the C1m and suvorexant groups as compared with the control group (Figure 6D), consistent with the previous observation that these cells only express OX_2_R (Mieda et al., 2011). Unexpectedly, we observed that the suvorexant group showed a larger number of Fos-IR orexin neurons in the LHA as compared with the control and C1m-treated groups (Figure 6A), although a previous study suggested that orexin neurons express OX_2_R (Yamanaka et al., 2010). The increased number of Fos-IR orexin neurons in the suvorexant group compared with the control group might have resulted from decreased activity of monoaminergic neurons, which were shown to send inhibitory feedback projections to orexin neurons (Sakurai and Mieda, 2011). Inhibitory feedback mechanisms mediated by noradrenergic neurons might play a major role in regulation of orexinergic activity, because C1m did not affect the number of Fos-IR neurons (Figure 6A). Alternatively, blockade by suvorexant of OX_1_R-mediated activation of GABAergic interneurons that send inhibitory projections to orexin neurons might increase the activity of orexin neurons (Matsuki et al., 2009). The suvorexant-mediated increase in orexin neuronal activity might be one of the possible reasons for the rebound wakefulness seen in suvorexant-administered mice in the latter half of the dark period after administration at ZT12 (Figure 2A).

To precisely compare the effects of DORA vs. 2-SORA on sleep/wakefulness states, it would be necessary to compare the effects at equal free brain concentrations and also to have data of brain receptor occupancy. Although we do not have such data, our present results would be useful for further understanding the characteristics of the effects of DORA and 2-SORA and the roles of the two orexin receptors in sleep/wakefulness regulation.

## Conclusion

Given the comparable values of % reduction of wakefulness time for 6 h after administration of C1m (30 mg/kg) and suvorexant (30 mg/kg), −17.2% [*F*_(3, 26)_ = 9.55, *p* < 0.01] and −17.8% [*F*_(2, 20)_ = 3.74, *p* < 0.05] (Figures 1A, 3A), respectively, C1m, a newly developed 2-SORA, sufficiently suppressed wakefulness and promoted sleep with comparable efficacy to that of suvorexant, a potent DORA. However, suvorexant induced more stable sleep with longer NREM sleep episode duration and fewer NREM to wakefulness transitions, suggesting that additional OX_1_R blockade confers more stable sleep. On the other hand, C1m showed little effect on REM sleep time while suvorexant significantly increased REM sleep time. These results suggest that the different effects of DORA vs. 2-SORA on orexin-target neurons might reflect differences in the effects of these two drugs on sleep/wake behavior in mice.

## Author contributions

Keishi Etori performed all experiments, and wrote the paper. Yuki Saito and Natsuko Tsujino performed the experiments. Takeshi Sakurai designed and supervised the experiments, and wrote the paper.

### Conflict of interest statement

The authors declare that the research was conducted in the absence of any commercial or financial relationships that could be construed as a potential conflict of interest.

## Supplementary material

Figure S1 **Hourly analysis of effects of C1m on basal sleep/wakefulness states in C57BL/6 mice (***n*** = **6**–9/group) after administration at ZT12**. C1m (10, 30, 90 mg/kg) and methylcellulose as control were administered *per os* at the start of the dark period (*t* = 0, ZT12). Total time spent in each state **(A–C)** and average episode duration of each state **(D–F)** over 24 h. Data for the dark and light periods are displayed with light gray and white backgrounds, respectively. ^*^*p* < 0.05 for 10 mg/kg C1m, ^+^*p* < 0.05 for 30 mg/kg C1m, ^#^*p* < 0.05 for 90 mg/kg C1m vs. control, two-way ANOVA followed by Bonfferoni correction as a *post-hoc* test. Results are expressed in minutes and presented as mean ± s.e.m (*n* = 9 for control, *n* = 6 for 10 mg/kg C1m, *n* = 9 for 30 mg/kg C1m, *n* = 6 for 90 mg/kg C1m).

Figure S2 **Hourly analysis of effects of C1m on basal sleep/wakefulness states in C57BL/6 mice (***n*** = **6**–7/group) after administration at ZT0**. C1m (10, 30, 90 mg/kg) and methylcellulose as control were administered *per os* at the start of the dark period (*t* = 0, ZT0). Total time spent in each state **(A–C)** and average episode duration of each state **(D–F)** over 24 h. Data for the dark and light periods are displayed with light gray and white backgrounds, respectively. ^*^*p* < 0.05 for 10 mg/kg C1m, ^+^*p* < 0.05 for 30 mg/kg C1m, ^#^*p* < 0.05 for 90 mg/kg C1m vs. control, two-way ANOVA followed by Bonfferoni correction as a *post-hoc* test. Results are expressed in minutes and presented as mean ± s.e.m (*n* = 7 for control, *n* = 7 for 10 mg/kg C1m, *n* = 7 for 30 mg/kg C1m, *n* = 6 for 90 mg/kg C1m).

Figure S3 **Hourly analysis of effects of suvorexant on basal sleep/wakefulness states in C57BL/6 mice (***n*** = **7**–9/group) after administration at ZT12**. Suvorexant (10, 30 mg/kg) and methylcellulose as control were administered *per os* at the start of the dark period (*t* = 0, ZT12). Total time spent in each state **(A–C)** and average episode duration of each state **(D–F)** over 24 h. Data for the dark and light periods are displayed with light gray and white backgrounds, respectively. ^*^*p* < 0.05 for 10 mg/kg suvorexant, ^+^*p* < 0.05 for 30 mg/kg suvorexant vs. control, two-way ANOVA followed by Bonfferoni correction as a *post-hoc* test. Results are expressed in minutes and presented as mean ± s.e.m (*n* = 9 for control, *n* = 7 for 10 mg/kg suvorexant, *n* = 7 for 30 mg/kg suvorexant).

Figure S4 **Hourly analysis of effects of suvorexant on basal sleep/wakefulness states in C57BL/6 mice (***n*** = **5**–7/group) after administration at ZT0**. Suvorexant (10, 30 mg/kg) and methylcellulose as control were administered *per os* at the start of the dark period (*t* = 0, ZT0). Time spent in each state **(A–C)** and average episode duration of each state **(D–F)** over 24 h. Data for the dark and light periods are displayed with light gray and white backgrounds, respectively. ^*^*p* < 0.05 for 10 mg/kg suvorexant, ^+^*p* < 0.05 for 30 mg/kg suvorexant vs. control, two-way ANOVA followed by Bonfferoni correction as a *post-hoc* test. Results are expressed in minutes and presented as mean ±s.e.m. (*n* = 7 for control, *n* = 7 for 10 mg/kg suvorexant, *n* = 5 for 30 mg/kg suvorexant).
