# Supplementary figures and images for: Effects of a newly developed potent orexin-2 receptor-selective antagonist, compound 1 m, on sleep/wakefulness states in mice
Source: Front Neurosci. 2014 Jan 31;8:8. doi: 10.3389/fnins.2014.00008 (PMC3907770; doi:10.3389/fnins.2014.00008)

—●— Control —○— 10 mg/kg —▲— 30 mg/kg —◆— 90 mg/kg

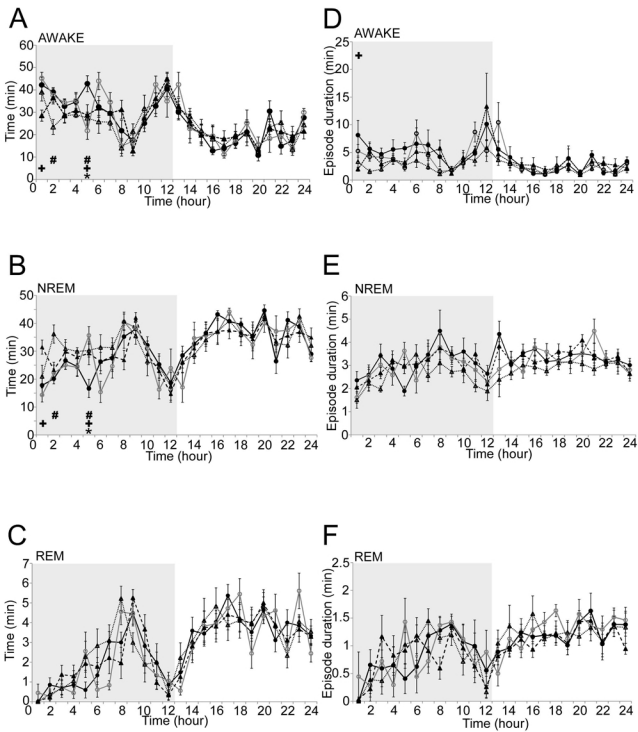

Supplementary fig. 1

Supplement: Figure S1 — Hourly analysis of effects of C1m on basal sleep/wakefulness states in C57BL/6 mice (n = 6–9/group) after administration at ZT12. C1m (10, 30, 90 mg/kg) and methylcellulose as control were administered per os at the start of the dark period (t = 0, ZT12). Total time spent in each state (A–C) and average episode duration of each state (D–F) over 24 h. Data for the dark and light periods are displayed with light gray and white backgrounds, respectively. *p < 0.05 for 10 mg/kg C1m, +p < 0.05 for 30 mg/kg C1m, #p < 0.05 for 90 mg/kg C1m vs. control, two-way ANOVA followed by Bonfferoni correction as a post-hoc test. Results are expressed in minutes and presented as mean ± s.e.m (n = 9 for control, n = 6 for 10 mg/kg C1m, n = 9 for 30 mg/kg C1m, n = 6 for 90 mg/kg C1m). [file Presentation1.PDF]

—●— Control —○— 10 mg/kg —▲— 30 mg/kg —◆— 90 mg/kg

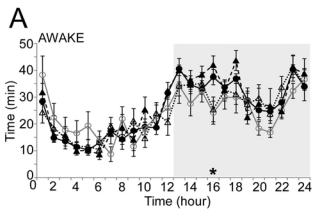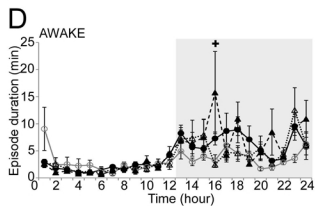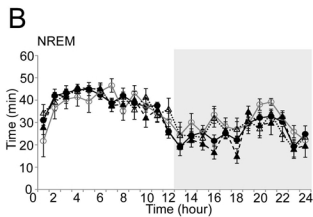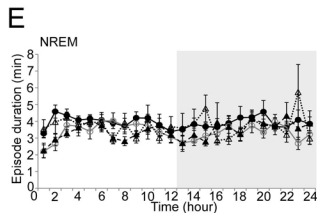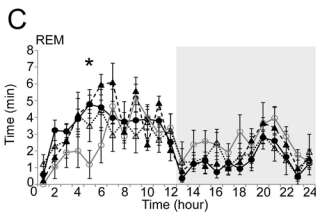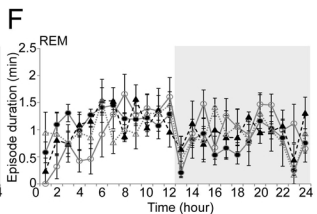

Supplement: Figure S2 — Hourly analysis of effects of C1m on basal sleep/wakefulness states in C57BL/6 mice (n = 6–7/group) after administration at ZT0. C1m (10, 30, 90 mg/kg) and methylcellulose as control were administered per os at the start of the dark period (t = 0, ZT0). Total time spent in each state (A–C) and average episode duration of each state (D–F) over 24 h. Data for the dark and light periods are displayed with light gray and white backgrounds, respectively. *p < 0.05 for 10 mg/kg C1m, +p < 0.05 for 30 mg/kg C1m, #p < 0.05 for 90 mg/kg C1m vs. control, two-way ANOVA followed by Bonfferoni correction as a post-hoc test. Results are expressed in minutes and presented as mean ± s.e.m (n = 7 for control, n = 7 for 10 mg/kg C1m, n = 7 for 30 mg/kg C1m, n = 6 for 90 mg/kg C1m). [file Presentation2.PDF]

● Control ○ 10 mg/kg ▲ 30 mg/kg

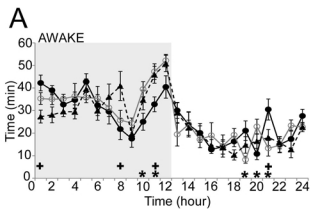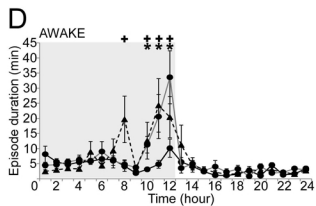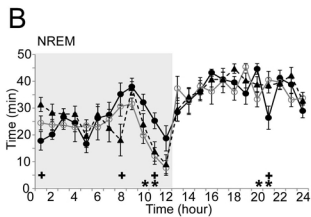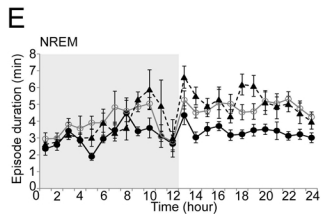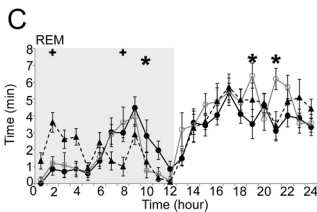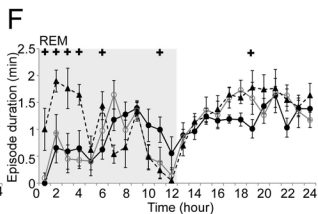

Supplement: Figure S3 — Hourly analysis of effects of suvorexant on basal sleep/wakefulness states in C57BL/6 mice (n = 7–9/group) after administration at ZT12. Suvorexant (10, 30 mg/kg) and methylcellulose as control were administered per os at the start of the dark period (t = 0, ZT12). Total time spent in each state (A–C) and average episode duration of each state (D–F) over 24 h. Data for the dark and light periods are displayed with light gray and white backgrounds, respectively. *p < 0.05 for 10 mg/kg suvorexant, +p < 0.05 for 30 mg/kg suvorexant vs. control, two-way ANOVA followed by Bonfferoni correction as a post-hoc test. Results are expressed in minutes and presented as mean ± s.e.m (n = 9 for control, n = 7 for 10 mg/kg suvorexant, n = 7 for 30 mg/kg suvorexant). [file Presentation3.PDF]

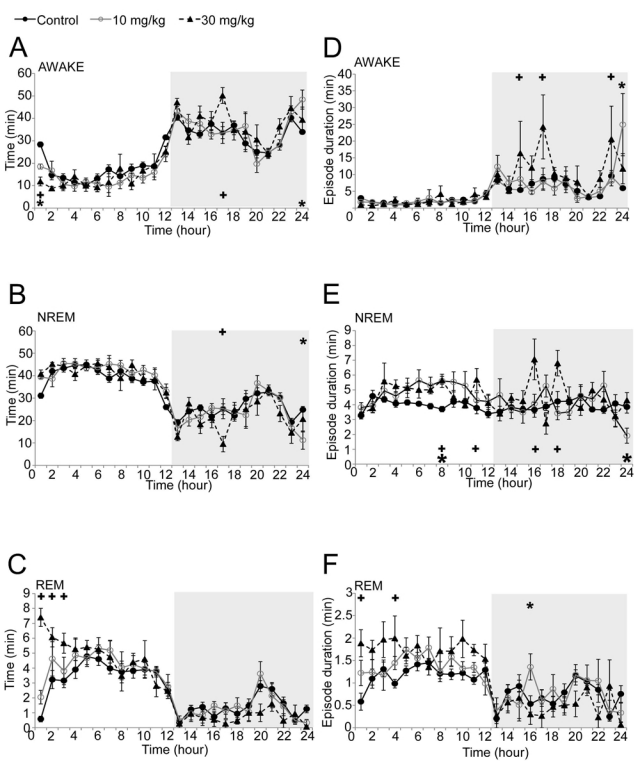

Supplementary fig. 4

Supplement: Figure S4 — Hourly analysis of effects of suvorexant on basal sleep/wakefulness states in C57BL/6 mice (n = 5–7/group) after administration at ZT0. Suvorexant (10, 30 mg/kg) and methylcellulose as control were administered per os at the start of the dark period (t = 0, ZT0). Time spent in each state (A–C) and average episode duration of each state (D–F) over 24 h. Data for the dark and light periods are displayed with light gray and white backgrounds, respectively. *p < 0.05 for 10 mg/kg suvorexant, +p < 0.05 for 30 mg/kg suvorexant vs. control, two-way ANOVA followed by Bonfferoni correction as a post-hoc test. Results are expressed in minutes and presented as mean ±s.e.m. (n = 7 for control, n = 7 for 10 mg/kg suvorexant, n = 5 for 30 mg/kg suvorexant). [file Presentation4.PDF]
